# Supplementary material for: Symbolic and non-symbolic numbers differently affect center identification in a number-line bisection task
Source: PLoS One. 2025 May 12;20(5):e0315654. doi: 10.1371/journal.pone.0315654 (PMC12068636; doi:10.1371/journal.pone.0315654)
Supplement: S1 Table — (DOCX) [file pone.0315654.s001.docx]

**S1. Post-Hoc results in Linear Mixed-Effects Models Results between flankers Numerosities and Format in Experiment 1**

| **Experiment 1** | | | | | | | |
| --- | --- | --- | --- | --- | --- | --- | --- |
| *Orientation* | *Format* | *Contrast* | *Emmean* | *Se* | *Df* | *Zratio* | *P.value* |
| Large-left | NON-SYM |  | -0.60 | 0.15 | Inf |  |  |
| Small-left | NON-SYM |  | -0.10 | 0.15 | Inf |  |  |
| Large-left | SYMB |  | -0.15 | 0.15 | Inf |  |  |
| Small-left | SYMB |  | -0.14 | 0.15 | Inf |  |  |
|  |  | (Large-left NON-SYM) - (Small-left NON-SYM) | -0.51 | 0.06 | Inf | -9.14 | **<.0001** |
|  |  | (Large-left NON-SYM) - Large-left SYMB | -0.46 | 0.06 | Inf | -8.20 | **<.0001** |
|  |  | (Large-left NON-SYM) - Small-left SYMB | -0.46 | 0.06 | Inf | -8.28 | **<.0001** |
|  |  | (Small-left NON-SYM) - Large-left SYMB | 0.05 | 0.06 | Inf | 0.95 | 0.78 |
|  |  | (Small-left NON-SYM) - Small-left SYMB | 0.05 | 0.06 | Inf | 0.87 | 0.82 |
|  |  | Large-left SYMB - Small-left SYMB | 0.00 | 0.06 | Inf | -0.08 | 0.99 |
