# Supplementary material for: Symbolic and non-symbolic numbers differently affect center identification in a number-line bisection task
Source: PLoS One. 2025 May 12;20(5):e0315654. doi: 10.1371/journal.pone.0315654 (PMC12068636; doi:10.1371/journal.pone.0315654)
Supplement: S2 Table — (DOCX) [file pone.0315654.s002.docx]

**S2. Post-Hoc results in Linear Mixed-Effects Models Results between flankers Numerosities and Format in Experiment 2**

| **Experiment 2** | | | | | | | |
| --- | --- | --- | --- | --- | --- | --- | --- |
| *Orientation* | *Format* | *Contrast* | *Emmean* | *Se* | *Df* | *Zratio* | *P.value* |
| Large-left | NON-SYM |  | -0.12 | 0.12 | Inf |  |  |
| Small-left | NON-SYM |  | -0.14 | 0.12 | Inf |  |  |
| Large-left | SYMB |  | 0.37 | 0.12 | Inf |  |  |
| Small-left | SYMB |  | 0.22 | 0.12 | Inf |  |  |
|  |  | (Large-left NON-SYM) - (Small-left NON-SYM) | 0.02 | 0.06 | Inf | 0.40 | 0.977 |
|  |  | (Large-left NON-SYM) - Large-left SYMB | -0.49 | 0.06 | Inf | -8.13 | **<.0001** |
|  |  | (Large-left NON-SYM) - Small-left SYMB | -0.34 | 0.06 | Inf | -5.65 | **<.0001** |
|  |  | (Small-left NON-SYM) - Large-left SYMB | -0.52 | 0.06 | Inf | -8.53 | **<.0001** |
|  |  | (Small-left NON-SYM) - Small-left SYMB | -0.36 | 0.06 | Inf | -6.05 | **<.0001** |
|  |  | Large-left SYMB - Small-left SYMB | 0.15 | 0.06 | Inf | 2.47 | 0.0638 |
