# Supplementary material for: Symbolic and non-symbolic numbers differently affect center identification in a number-line bisection task
Source: PLoS One. 2025 May 12;20(5):e0315654. doi: 10.1371/journal.pone.0315654 (PMC12068636; doi:10.1371/journal.pone.0315654)
Supplement: S3 Table — (DOCX) [file pone.0315654.s003.docx]

**S3. Post-Hoc results in Linear Mixed-Effects Models Results between flankers Numerosities and Format in Experiment 3**

| **Experiment 3** | | | | | | | |
| --- | --- | --- | --- | --- | --- | --- | --- |
| *Orientation* | *Format* | *Contrast* | *Emmean* | *Se* | *Df* | *Zratio* | *P.value* |
| Large-left | NON-SYM |  | -0.62 | 0.15 | Inf |  |  |
| Small-left | NON-SYM |  | -0.31 | 0.15 | Inf |  |  |
| Large-left | SYMB |  | -0.03 | 0.15 | Inf |  |  |
| Small-left | SYMB |  | -0.22 | 0.15 | Inf |  |  |
|  |  | (Large-left NON-SYM) - (Small-left NON-SYM) | -0.31 | 0.06 | Inf | -5.17 | **<.0001** |
|  |  | (Large-left NON-SYM) - Large-left SYMB | -0.58 | 0.06 | Inf | -9.72 | **<.0001** |
|  |  | (Large-left NON-SYM) - Small-left SYMB | -0.40 | 0.06 | Inf | -6.60 | **<.0001** |
|  |  | (Small-left NON-SYM) - Large-left SYMB | -0.27 | 0.06 | Inf | -4.56 | **<.0001** |
|  |  | (Small-left NON-SYM) - Small-left SYMB | -0.08 | 0.06 | Inf | -1.43 | 0.4758 |
|  |  | Large-left SYMB - Small-left SYMB | 0.18 | 0.06 | Inf | 3.12 | **0.0096** |
