# Supplementary material for: Symbolic and non-symbolic numbers differently affect center identification in a number-line bisection task
Source: PLoS One. 2025 May 12;20(5):e0315654. doi: 10.1371/journal.pone.0315654 (PMC12068636; doi:10.1371/journal.pone.0315654)
Supplement: S4 Table — (DOCX) [file pone.0315654.s004.docx]

**S4. Post-Hoc results in Linear Mixed-Effects Models Results between flankers Numerosities and Format in Experiment 4**

| **Experiment 4** | | | | | | | |
| --- | --- | --- | --- | --- | --- | --- | --- |
| *Orientation* | *Format* | *Contrast* | *Emmean* | *Se* | *Df* | *Zratio* | *P.value* |
| Large-left | NON-SYM |  | -0.44 | 0.16 | Inf |  |  |
| Small-left | NON-SYM |  | 0.002 | 0.16 | Inf |  |  |
| Large-left | SYMB |  | -0.007 | 0.16 | Inf |  |  |
| Small-left | SYMB |  | -0.24 | 0.16 | Inf |  |  |
|  |  | (Large-left NON-SYM) - (Small-left NON-SYM) | -0.45 | 0.06 | Inf | -7.03 | **<.0001** |
|  |  | (Large-left NON-SYM) - Large-left SYMB | -0.44 | 0.06 | Inf | -6.87 | **<.0001** |
|  |  | (Large-left NON-SYM) - Small-left SYMB | -0.20 | 0.06 | Inf | -3.13 | 0.0093 |
|  |  | (Small-left NON-SYM) - Large-left SYMB | 0.009 | 0.06 | Inf | 0.15 | 0.9987 |
|  |  | (Small-left NON-SYM) - Small-left SYMB | 0.24 | 0.06 | Inf | 3.9 | **0.0006** |
|  |  | Large-left SYMB - Small-left SYMB | 0.23 | 0.06 | Inf | 3.74 | **0.001** |
