# Supplementary material for: Symbolic and non-symbolic numbers differently affect center identification in a number-line bisection task
Source: PLoS One. 2025 May 12;20(5):e0315654. doi: 10.1371/journal.pone.0315654 (PMC12068636; doi:10.1371/journal.pone.0315654)
Supplement: S5 Table — B. Mean and Standard Deviations of each Experiment by Format and Orientation. (DOCX) [file pone.0315654.s005.docx]

**S5A. Mean and Standard Deviations of each Experiment combining format and orientation**

|  | *Exp 1* | | *Exp 2* | | *Exp 3* | | *Exp 4* | |
| --- | --- | --- | --- | --- | --- | --- | --- | --- |
| Condition | *Mean* | *DS* | *Mean* | *DS* | *Mean* | *DS* | *Mean* | *DS* |
| Baseline | -0.59 | 1.39 | -0.57 | 1.37 | -0.41 | 1.40 | -0.81 | 1.33 |
| Symbolic - Small-left | -0.14 | 1.61 | 0.22 | 1.66 | -0.22 | 1.62 | -0.24 | 1.79 |
| Symbolic - Large-left | -0.14 | 1.62 | 0.36 | 1.58 | -0.04 | 1.64 | -0.003 | 1.80 |
| Non-Symbolic - Small-left | -0.09 | 1.41 | -0.15 | 1.51 | -0.31 | 1.63 | 0.002 | 1.71 |
| Non-Symbolic - Large-left | -0.60 | 1.51 | -0.12 | 1.49 | -0.63 | 1.67 | -0.44 | 1.68 |

**S5B. Mean and Standard Deviations of each Experiment by Format and Orientation**

|  | |  | *Exp 1* | | *Exp 2* | | *Exp 3* | | *Exp 4* | |
| --- | --- | --- | --- | --- | --- | --- | --- | --- | --- | --- |
| Condition | |  | *Mean* | *DS* | *Mean* | *DS* | *Mean* | *DS* | *Mean* | *DS* |
| Format | Non-Symbolic |  | -0.35 | 1.49 | -0.14 | 1.51 | -0.47 | 1.66 | -0.22 | 1.71 |
|  | Symbolic |  | -0.15 | 1.62 | 0.30 | 1.63 | -0.14 | 1.64 | -0.12 | 1.80 |
| Orientation | Small-left |  | -0.12 | 1.52 | 0.04 | 1.60 | -0.27 | 1.63 | -0.12 | 1.76 |
|  | Large-left |  | -0.38 | 1.59 | 0.12 | 1.56 | -0.34 | 1.69 | -0.23 | 1.76 |
