# Supplementary material for: Symbolic and non-symbolic numbers differently affect center identification in a number-line bisection task
Source: PLoS One. 2025 May 12;20(5):e0315654. doi: 10.1371/journal.pone.0315654 (PMC12068636; doi:10.1371/journal.pone.0315654)
Supplement: S6 Table — T-test was performed to compare baseline with format and orientation combined, based on the subjects’ means. (DOCX) [file pone.0315654.s006.docx]

**S6. T-test baseline * (format+condition)**

T-test was performed to compare baseline with format and orientation combined, based on the subjects' means.

| baseline vs | EXP 1 | EXP 2 | EXP 3 | Exp 4 |
| --- | --- | --- | --- | --- |
| Small-left Non-Symbolic | t(33) = -3.09; p = 0.004 | t(33) = -2.86; p = 0.007 | t(33) = -0.50; p = 0.614 | t(33) = -3.72; p = 0.0007 |
| Large-left  Non-Symbolic | t(33) = 0.04; p = 0.962 | t(33) = -2.72; p = 0.010 | t(33) = 1.02; p = 0.314 | t(33) = -1.59; p = 0.12 |
| Small-left  Symbolic | t(33) = -2.47; p = 0.184 | t(33) = -4.39; p = 0.0001 | t(33) = -1.01; p = 0.317 | t(33) = -3.04; p = 0.004 |
| Large-left  Symbolic | t(33) = -2.41; p = 0.021 | t(33) = -5.31; p < 0.0001 | t(33) = -2.02; p = 0.051 | t(33) = -4.29; p = 0.0001 |
