# Supplementary material for: Symbolic and non-symbolic numbers differently affect center identification in a number-line bisection task
Source: PLoS One. 2025 May 12;20(5):e0315654. doi: 10.1371/journal.pone.0315654 (PMC12068636; doi:10.1371/journal.pone.0315654)
Supplement: S7 File — (DOCX) [file pone.0315654.s007.docx]

**S7. ANOVA (format x orientation x line length)**

In order to complement the LMMs and increase the robustness and generalisability of our conclusions, analysis of variance (ANOVA) was performed on the subjects' means, including line length, orientation and format as a factor as within-subject factors and participant as a random factor. The analysis was performed using the rstatix package in R.

Exp 1

ANOVA analyses confirmed a significant effect of orientation (F(1, 33) = 42.12, p < .001), format (F(1, 33) = 2.49, p = .12), line length (F(1, 33) = 0.87, p = .35), the interaction between orientation and format (F(1, 33) = 50.94, p = < .001), the interaction between orientation and line length (F(1, 33) = 0.001, p = .97), the interaction between format and line length (F(1, 33) = 7.87, p = .008), and the interaction between orientation, format, and line length (F(1, 33) = 1.27, p = .26).

Exp2

ANOVA analyses confirmed a significant effect of orientation (F(1, 33) = 7.65, p = .009), format (F(1, 33) = 11.84, p = .002), line length (F(1, 33) = 2.99, p = .09), the interaction between orientation and format (F(1, 33) = 3.38, p = .07), the interaction between orientation and line length (F(1, 33) = 1.38, p = .24), the interaction between format and line length (F(1, 33) = 0.26, p = .61), and the interaction between orientation, format, and line length (F(1, 33) = 0.59, p = .44).

Exp3

ANOVA analyses confirmed a significant effect of orientation (F(1, 33) = 2.44, p = .12), format (F(1, 33) = 8.32, p < .001), line length (F(1, 33) = 4.27, p = .04), the interaction between orientation and format (F(1, 33) = 32.70, p < .001), the interaction between orientation and line length (F(1, 33) = 0.04, p = .83), the interaction between condition and line length (F(1, 33) = 3.65, p = .06), and the interaction between orientation, format, and line length (F(1, 33) = 0.01, p = .89).

Exp4

ANOVA analyses confirmed a significant effect of orientation (F(1, 33) = 5.92, p = .02), format (F(1, 33) = 0.45, p = .50), line length (F(1, 33) = 0.42, p = .51), the interaction between orientation and format (F(1, 33) = 49.74, p < .001), the interaction between orientation and line length (F(1, 33) = 0.25, p = .62), the interaction between format and line length (F(1, 33) = 6.81, p = .01), and the interaction between orientation, format, and line length (F(1, 33) = 1.49, p = .23).
