# Supplementary material for: Symbolic and non-symbolic numbers differently affect center identification in a number-line bisection task
Source: PLoS One. 2025 May 12;20(5):e0315654. doi: 10.1371/journal.pone.0315654 (PMC12068636; doi:10.1371/journal.pone.0315654)
Supplement: Attachment — Submitted filename: Response to Reviewers.docx [file pone.0315654.s009.docx]

March 10th, 2025

Elisa Scerrati, Ph.D.  
Academic Editor

PLOS ONE

Dear Prof. Scerrati,

Thank you very much for your consideration of our manuscript and for the opportunity to submit a revised version of it. We appreciate the time and effort that the reviewers have dedicated to providing valuable comments. We have engaged in the revisions based on the two reviewers, with particular attention to providing justification to the methods and extending the introduction and discussion, as suggested. Moreover, we have directly addressed all the concerns raised and included a point-by-point response of each below. All the corresponding changes have been highlighted in blue in the manuscript.

We believe that this revision has substantially clarified and improved our manuscript. We hope that you and the reviewers agree and consider accepting this manuscript for publication in PLOS ONE

Yours sincerely,

The authors

**RESPONSE TO REVIEWER 1**

**0. Let me begin by providing relevant context that is missing from this manuscript.**

**The line bisection task, naively adopted by neuropsychologists for efficient bed-side diagnostics (like other seemingly simple tasks, e.g., Corsi blocks), has long been studied to understand sensory, cognitive, and motor contributions to performance. Perceptual flankers were added to manipulate visual attention prior to bisection by demanding their verbal reporting.**

**Ever since, perceptual and attentional contributions to flanked line bisection performance were discussed, but I have never encountered a discussion of conceptual representations in the context of flanker manipulations. This brings me to my first (conceptual) problem.**

**1. The objectives and methods seem misaligned. The main effect of the number flanker format is taken to argue for distinct representations (e.g., line 327f, 402f). But the bisection task measures sensory/perceptual (and also motor) but not conceptual factors. This is evident in all four results (largest stimulus extent on the left creates largest left bias) and in the wider literature. Thinking of the bisector placement as the participant’s subjective balance point of a scale (the line), what can the effect of placing a picture of an elephant or ant vs the word elephant/ant reveal about the cognitive representation of elephants? Results will depend on the font size, picture size, etc. Consistent with this assessment, the present report identifies perceptual grouping effects, as did Fischer (1994). This study should be added to the review and the interpretation of results adjusted.**

**Fischer MH. Less attention and more perception in cued line bisection. Brain Cogn 1994;25:24–33**

We thank the Reviewer for this insightful comment, which captures the essence of the study. Although the line bisection task has been widely used in studies of cognitive psychology and neuropsychology, whether biases result from perceptual, attentional or conceptual factors is still a matter of controversy, particularly in the field of numerical cognition. Crucially, previous studies found that, even in the absence of explicit cueing of the flankers or requests for verbal reports of them, healthy adult participants would manifest a “conceptual” representation of flankers (i.e. magnitudes) compatible with the organization over a mental number line. The authors also found that this representation is abstract, insofar as it extends to symbolic and non-symbolic formats. The present study attempts to replicate such findings, and to extend them by manipulating perceptual cues in the original stimuli. As it turns out, these manipulations converge on the relevance of perceptual cues more than on its abstract representations.

In the revised version of the manuscript, we have exposed these controversies in the introduction to provide the reader with a more comprehensive view of the literature (p 3; lines 68). In doing that, the study by Fischer (1994) becomes relevant, along with other studies. We thus thank the reviewer for this suggestion.

**1. Turning to methodological issues, I believe statistics can be improved. Effects should be computed relative to the baseline bisection not relative to zero.** **ANOVA should (also) be performed on subject means (as was the t-test, i.e. with 33 degrees of freedom). Importantly, the factor line length (cf line 211) should be included to reduce error variance and increase effect sizes (over which the authors complain in line 388).**

We thank the reviewer for these suggestions.

Let us first clarify that most of our methodological decisions were made with the aim of providing a common framework to make meaningful comparisons between ours and previous studies. The decision to report the deviations compared to the objective midline is one of them. To the best of our knowledge, there is no study in the literature of number processing that reports and discusses the effects in relation to the baseline (subjective performance). Conversely, all of them report the results relative to zero (objective midline). Still, we acknowledge that small deviations in marking the midpoint may result from motor variability across participants. Thus, the requested comparisons are now included in the Supplementary Materials.

Similarly, the factor line length -agreeably relevant for the task (but see contradictory results in the metaanalysis of Jewell & McCourt, 2000)- was manipulated but not analyzed in studies in which the present study was built upon (i.e., DeHevia & Spelke, 2009). However, acknowledging the Reviewer’s suggestion, we have included line length as a factor in an ANOVA performed on subject means, as requested. The results of this new set of analyses are also included in the Supporting Information (p 33-35).

**2. The literature review is suboptimal because of unclear formulations (see my many minor comments below). Readers would benefit from stating explicitly the two main effect patterns reported in this literature, namely a tendency to orient towards larger surface flankers and towards larger symbolic magnitudes.**

We thank the reviewer for allowing us to clarify these points. We have reformulated the text in the introduction and explicitly inform the readers about the two most relevant and originally expected patterns (p 3, lines 68-70).

**3. Hypotheses should also be stated more clearly. For Experiment 1, the expectation is not “similar results” but “similar bias towards the larger number”. For Experiment 2 the hypothesis is not accurate bisection because of the extensive evidence for pseudo-neglect, which should be explained better and explicitly credited to Jewell & Mc Court (2000) in line 82 as part of the literature review, and again in lines 130 and 144.**

The new version of the manuscript implements a reformulation in an attempt to make the research hypotheses more explicit (p 4, lines 25), as suggested by you and by Reviewer 2.

A definition of the pseudoneglect and corresponding reference to Jewell & McCourt (2000) has been also included.

**4. I was surprised to see the study was conducted on paper (136 participants x 132 trials with single lines per page = 17,952 sheets of paper!). Since several of the experiments reviewed as background were conducted on computer displays, the authors should justify their decision and discuss whether the medium of testing might have contributed to the alleged heterogeneity of results (in the Intro) as well as to their own findings (in the Discussion). Luh et al. (1995) is an example of a comparative bisection study.**

**Luh, K. E. (1995). Line bisection and perceptual asymmetries in normal individuals: What you see is not what you get. Neuropsychology, 9(4), 435–448.** [**https://doi.org/10.1037/0894-4105.9.4.435**](https://doi.org/10.1037/0894-4105.9.4.435)

As explained above, the methodological implementations of the current studies were designed to be equivalent to previous studies evaluating the effects of numerical flankers on the line bisection. To the best of our knowledge, there are eight studies (out of ten) that used paper-and-pencil in this context. We thus deputed this modality not only as the most conventional but also comparable with previous experimental manipulations. This information has been included in the text (p. 10, lines 240,241). This is not to deny that other implementations could be more precise. Possible limitations of this modality have been now acknowledged in the discussion (p. 19-20, lines 464-468).

We also thank the reviewer for pointing out the article by Luh (1995), which was now included in the discussion regarding possible implications of this methodological choice.

**5. The authors feel compelled to invoke left-lateralized cortical symbol processing (lines 341 ff) to account for right-bias with symbolic numbers. First, this incomplete argument should be augmented by explaining to readers (a) the cross-lateralization of visual processing, (b) the relation between hemispheric activity and attention deployment, and (c) the link between attention deployment and subjective line extent. Secondly, the “right bias” is only a relative bias, depending on baseline performance (which incidentally depends on line length). The same relativity holds for “left bias”, which is on top of the hemi-neglect bias. Awareness of this compound nature of bisection effects should inform the interpretation.**

We appreciate the reviewer’s request for additional information. A more extended explanation of the phenomenon has been now included in the discussion (p 17-18, lines 407-419).

Minor issues

**Line 68: the subitizing range is irrelevant for symbolic number processing, so the authors need to clarify that the study used non-symbolic numbers. Moreover, 9 is not within the subitizing range.**

Due to numerous modifications, this passage has been erased from the current version of the manuscript. We have modified the previous sentence accordingly (also based on Reviewer’s 2 suggestions) and integrated this information in a different section of the manuscript (p. 3,5). We do not mention the subitizing range in this new report (which originally referred to one of the flankers i.e., 2, not to both).

**Line 80: the authors should describe the number position effect in full to be more useful for the readers. In general, the entire description can be improved (e.g., line 83: near or far from what?**

The line position effect has been described in a more specific way (lines 71-79). Moreover we have attempted a more accurate description of the studies.

**Line 81: what is a lateral disparity?**

We meant illusory spatial extension. In line 95, we modified the phrase accordingly 127-130

**Line 99: what is the comparison task?**

This phrase has been erased in the current version of the manuscript.

**Line 110: closer to what?**

Closer to the midline point. We have changed it to “more accurately”, to clarify it. Line 116-118.

**Line 146: the expression “denser in the line proximity” contains two aspects (density and proximity) – please clarify.**

The phrase has been modified in the following way: “larger numbers or larger quantities closer to the line” (lines 147-157).

**Line 166: Was handedness self-reported or measured?**

It was self-reported. This has been clarified in the text, line 176.

**Line 182: Clarify whether the baseline was always the first four trials (as implied by the word “phase”) or whether these were within the four blocks.**

Yes. The reviewer is right. The baseline was always presented at the beginning of each experiment. We have added this information in line 247.

**Line 209: what is “gsm” and which part of a stimulus was printed in white (assuming the pages were white)?**

GSM stands for 'grams per square metre' and refers to the weight of the paper. The heavier the paper, the ticker and the higher the number of grams per square metre. The stimuli was black, printed on a white paper. This has been corrected in lines 185-186.

**Line 201: Lines were centered on the page –inviting use of the page as spatial reference and potentially reducing experimentally induced effects. The authors might want to acknowledge this.**

Thanks for this suggestion. The information has been added in lines 199-201.

**Line 220: this is the only mention of an “invisible square” – what was its purpose etc? I assume the answer will also clarify in what sense flanker digits for horizontally displayed lines can be horizontally centered” (would this not mean they appear above the line?).**

The stimuli were presented within an invisible square of 5cmx5cm that served as the framework for the accurate location of the digit across trials and conditions. The digit was vertically centered with respect to the square and horizontally aligned towards the line. The line was centered vertically, with the digits positioned equidistant from both ends. This information has been added in the text (line 195, 199-202).

**Line 262: Report baseline mean and add the unit mm to all means.**

We are indebted to the suggestion for prompting us to report the mean and standard deviation of the baseline for each experiment. We have also incorporated unit measures into all descriptive analyses.

**Line 284: this sentence is incomplete.**

Thanks. This was a typo. The sentence we deleted.

**RESPONSE TO REVIEWER 2**

**The manuscript (PONE-D-24-53823) presents a Research Article treating four line-bisection experiments which feature different kinds of numerical flankers, either symbolic or non-symbolic in format. Stronger leftward biases on line bisection are found in the non-symbolic format, leading the authors to conclude that processes akin to pseudoneglect might account for this; whereas the rightward biases encountered in the symbolic format are said to reflect an organized representation of numbers.**

**While the manuscript is well written, the design carefully thought and statistical analyses adequately done, I have some major concerns that should in my opinion be addressed before proceeding with the publication process. Therefore, my suggestion is to carry out a major revision.**

**1. I have some doubts about the methodology – detailed below – which do not necessarily question the soundness of the study but that should be addressed, nonetheless.**

The concerns regarding the methodology have been addressed below, following each specific comment (lines 62-70).

**2. The manuscript is overly brief when detailing the experimental manipulations and their operational relevance to test the study’s hypothesis.**

We thank the reviewer for this insight. The new version of the manuscript implements a reformulation in an attempt to make the research hypotheses more explicit and the experimental manipulations clearer (p 6-7).

**3. I did not see a great effort in discussing the two main results, which crucially differ depending on the flanker’s format (left/right-ward biases for symbolic/non-symbolic), jointly. In the end, each of them is traced back to theoretical explanations that are not novel (i.e., pseudoneglect and scanning habits). While this is not bad in principle, I think the great work carried out to design this study deserves to be discussed in little more depth.**

In this new version we have modified the discussion to highlight possible mechanisms behind the differences obtained with the two formats. Specifically, the influence of attentional deployment over the flankers depending on the saliency (and perceived line length) has been discussed, particularly relevant for the non-symbolic format. The interplay between the lateralization of the brain mechanisms involved in spatial attention and Arabic number processing has been also considered to account for opposite results in the symbolic format (lines 382-399).

**Detailed comments**

**• Line 61-62: this statement is quite generic and seems unnecessary.**

In this version of the manuscript we have attempted to develop better the connections between evolutionary number space associations and the mature forms of the mental number line (lines 58-64).

• **Lines 65 – 66: what do the leftward and rightward biases substantiate in?**

The results were explained in terms of the mental number line hypothesis which, in the new version of the manuscript, has been better explained in the introduction, before the results of Fischer's study (lines 71-78).

**• Line 80: the results from this experiment are not clear. It is particularly not clear what “the position of the strings on the sheet” means.**

This has been rephrased in the current version. Findings of the study suggested the effects of numbers in the task are better explained in terms of spatial reference frames (i.e., whether the line was placed in the left, right or central position of the page).

• **Lines 76 – 91: I do not think that dedicating an entire paragraph to one single study is justified here, especially because it comprises many studies, the results of which cannot be addressed in detail for obvious reasons. I suggest to briefly outline the rationale behind the study and skipping to the general conclusion as at lines 89 – 91.**

We thank the reviewer for this suggestion. In the new version of the manuscript we have summarized the main results of the study (lines 87-96).

• **Lines 110 – 111: the relationship between Gebuis and Gevers’ and the claim that introduces the paragraph (“distinction between symbolic and non-symbolic numbers’ representations” – line 106) is not clear.**

Thanks a lot for this suggestion. Gebuis and Gever's study highlights how weightening different visual cues could hinder or influence the extraction of numerosity information, affecting the automatic associations between numbers and space and, in turn, the direction of the biases. The influence of visuo-perceptual cues is not necessarily expected for symbolic representations, which somehow questions the abstract mapping hypothesis. This information has been now included in the text (lines 113-121) .

**• Lines 185 – 187: “Moreover … of the line”. This is not clear. What is the most “prominent” flanker? Why would it be placed on the left side of the line only? Please articulate.**

Thanks for prompting us to clarify this. To better explain the experimental condition, we have rephrased in the following way:

*“Furthermore, when asymmetrical, flankers could be arranged so that either the most prominent (Large-left) or the least prominent (Small-left) element appears on the left side of the line”.* (lines 251-256).

• **Figure 1 is in low resolution; moreover, it is overly concise. I think splitting it into two figures would make more sense.**

We have reworked the image, which should now have a better resolution. We decided to keep a single display because we believe it gives a general overview of all the results for comparative analysis. However, if more information is required and the image becomes too crowded or unclear, we are willing to modify the display.

**• Lines 221 – 236: referencing to the figure and panel would help. Please also add a title for the very first column in figure 1 panel a – in which are the definitions Baseline, Small-left, Large-left…**

We appreciate the reviewer's suggestion, we have added the title and also the panel labels for the images and the title for the first column in panel a, which presents the orientation conditions.

**• Lines 221, 223, 226 and any other occurrence: Please be consistent when referencing to the flankers: either use flankers, flanker stimuli or stimuli.**

We have harmonised the text by indicating everything with flanker stimuli (p. 9).

• **Lines 223 – 224: “The stimuli “2-2” were arbitrarily considered the Small-left orientation and the “8-8” were considered the Large-left orientation” I do not understand this arbitrary choice, more so because I did not understand the rationale for this manipulation as explained at lines 185 – 187.**

Thanks. We hope that, by explaining the rationale of the study (as per your comment above), this information becomes clear.

Additionally, for statistical comparisons across studies we used two factors (orientation and format), and given that identical stimuli in Experiment 2 lacked orientation (they were symmetrical), we arbitrarily designated “2-2” as the Small-left orientation and “8-8” as the Large-left orientation, to keep the statistical models constant across Experiments. Notably, no main effects of orientation or interactions were found in this study, as expected. This information has been now added to lines 254-256.

**• Lines 332 – 335 “The behaviour in the Non-symbolic format is compatible with pseudoneglect” Was the leftward bias at all present in the symbolic format, though less intense? If that is the case, then I suggest rephrasing.**

Yes, the reviewer is right. We clarify that sometimes there was a right bias, and sometimes a less intense leftward bias was found (lines 407-412). Possible explanations of this effect were also outlined in the discussion.

**• Lines 357 – 364: I found this paragraph quite confusing, particularly so because there is an overlap between the terminology used by the authors (i.e., “scanning from left-to-right”) and what we usually refer to when talking about cultural-linguistic scanning. The authors should clarify if they are treating a cultural habit or a situational behaviour. In the latter case, I still believe the cultural bias for that should at least be acknowledged.**

In the new version of the paper, we have clarified the cultural scanning aspect by formally acknowledging the importance of attentional and perceptual factors in a line bisection task, even when numerical information is used (p. 18 lines 418-438).

*“It is thus possible that participants adopt a bisection strategy that systematically scans from left to right, linked to cultural bias (e.g. reading direction), but when cue oriented in a different direction (due to saliency disparities of the flankers) they adopt a situational behavior, displaying an opposite bisection pattern.”*

**• Lines 368 – 369: “a right-to-left direction bias possibly counteracts the pseudoneglect phenomenon, yielding responses closer to the midpoint”. This was addressed a little too superficially. What would motivate such a bias?**

Thanks, a more detailed explanation has been offered. The following paragraph has been added (lines 439-444):

*“the leftward responses consequent of pseudoneglect were enhanced when the larger flankers were on the left (i.e. Large-left orientation). This is likely due to the deployment of attention to the most prominent flanker and the consequent left-to-right scanning over the line. Conversely, when the larger flankers were on the right (i.e. Small-left orientation) and attention was directed accordingly (inducing right-to-left scanning), the pseudoneglect phenomenon was counteracted, yielding responses closer to the midpoint.”*

**General comments**

**• Procedure – this part could go into little more detail. How long were the lines?**

Line 187: The line was 1 mm wide, the length was either 60 or 80 mm and it was counterbalanced across trials.

**How big were the flankers?**

The exact dimensions of the flankers are specified in the revised version of the manuscript, p 8-9.

**What were the numerosity of the flankers (both for symbolic and non-symbolic)**

These details are specified in the revised version of the manuscript, p 8-9.

**How long on average did it take to hand the single sheets?**

We regret that we are unable to fully address the reviewer’s request, as we did not record the exact time of the administration.

**Why was a pencil-and-paper version choosing instead of a digital one?**

We thank the author for allowing us to clarify this aspect; our study was designed to be equivalent to previous studies that have evaluated the effects of numerical flankers on line bisection. To the best of our knowledge, there are eight studies (out of ten) that have used paper and pencil in this context. We therefore considered this method to be not only the most conventional, but also comparable to previous experimental manipulations. This information has been included in the text (lines 240-241).

**• *Edit* - while continuing reading, I realized the details missing in the procedure are actually in the Stimuli and Materials. I am leaving my prior comment because readers could get confused like me for not being able to read these details before the procedure. Please consider switching the two sections (Stimuli first – procedure second)**

We thank the reviewer for suggesting. We have now moved the description of the stimuli before the procedure (p 8-10).

• **I think a further justification for the paper-and-pencil methodology other than citing De Hevia and Spelke is needed. This choice leaves me puzzled as I believe precision in collecting and coding data has been undermined by this choice. How have potential biases or human errors been prevented? For instance, I am worried that letting participants carry the bisection manually could have let them adjust their hand position as to securely bisect it more or less at the same spot. Please elaborate on this.**

As mentioned above, we chose to use this designed to be equivalent to previous research. However, we fully agree with the reviewer that each methodology may lead to different measurement errors. We have therefore included this aspect as a limitation of our research (lines 466-468).

**• I would have appreciated a very brief introduction and/or discussion section for each experiment, summarizing the conditions in focus and the relevance of the results from each experiment for the hypothesis that informed its design. It would help focus the relevance of each experiment for the general research question.**

We have added a brief introduction in the discussion for each experiment (lines 382-399).

**Typos and minor concerns**

• Line 73: “systematically bias” biases [changed]

• Line 192: You used “numerosity 8” and “numerosity two”. Please homologate the format. [changed]

• Line 284: “Moreover, deviations from the objective center of the line.” I guess this sentence is incomplete. [changed]
